# Supplementary material for: Inorganic Carbon Assimilation and Electrosynthesis of Platform Chemicals in Bioelectrochemical Systems (BESs) Inoculated with Clostridium saccharoperbutylacetonicum N1-H4
Source: Microorganisms. 2023 Mar 13;11(3):735. doi: 10.3390/microorganisms11030735 (PMC10051846; doi:10.3390/microorganisms11030735)
Supplement: Supplementary file 1 [file microorganisms-11-00735-s001.zip › microorganisms-2130638-supplementary.pdf]

SUPPLEMENTARY MATERIALS

S.1. Methological approach to bioelectrodes formation

It is well known how actively growing cultures can form biofilms on both biotic and abiotic surfaces, and how this property can be used to study the ability of bacteria to form biofilms and to interact with other microorganisms [1][2][3]. Among other factors, the density of the starting microbial culture, medium composition, surface charge density, as well as wettability and structure of the surface affect biofilm formation and architecture [1][2]. It has been demonstrated that the use of dynamic (with medium flowing), and even semi-dynamic conditions (i.e., in shaking conditions), can enhance biofilm formation by increasing the EPS production and strength of the EPS matrix, providing nutrients for growth, and promoting expression of molecules involved in signal transduction, as demonstrated in *P. aeruginosa* [1][2]. This results in the generation of denser and thicker biofilms able to resist environmental stressors, even in multispecies biofilms [1]. As our experimental scheme envisaged the gradual change of culture media along with the biofilm formation (from complex to minimal formulations), we periodically checked the electrodes potentials and the oxidoreductive potential (ORP) of non-BES control cultures vs. Ag/AgCl reference electrodes to collect information about the change in their redox state and, indirectly, of microbial metabolism [3].

In fact, in culture media, ORP correlates the net balance of intracellular reducing equivalents, which is involved in protein, DNA and RNA synthesis, enzyme activation, and even regulation of the cell cycle. Thus, monitoring and controlling environmental redox potential helps to elucidate cellular physiology and intracellular metabolic interaction. For this reason, ORP is widely used to monitor fermentation processes, control microbial metabolism, and improve the production of target molecules [3].

A prerequisite for CO_2 uptake by bacteria is its dissolution in the culture medium, forming firstly H_2CO_3, which rapidly converts to HCO_3^- + H^+. At saturation, an equilibrium is established between CO_2 and HCO_3^-. We then used $NaHCO_3$ during the preparation of biocathode instead of gaseous CO_2, as the presence of bubbles in the medium might have affected the microbial adhesion to the electrode surface. Furthermore, by connecting the carbon cloth electrodes with a 100 Ohm resistor, we aimed first at distancing the electrodes from each other and then detecting the presence of any significant current flowing between them due to the establishment of different values of electrochemical potentials, which might have led to significant differences in biofilms. We used this approach to try to balance the fermentative metabolism of Clostridium cells growing in the medium. Proper analyses are needed to investigate the effects of the snorkel on the electrochemical properties of *C.saccharoperbutylacetonicum* biofilms.

S2. Dissolved CO_2 calculations

As a first step, for each system, we calculated CO_2 assimilation rate, according to Eq 1S:

$$\boldsymbol{CO_{2ass}} = \left[\frac{\mathbf{CO2\ in - CO2ev}}{\mathbf{CO2in}}\right] \mathbf{x\ 100} \qquad \text{Eq (1S)}$$

where CO_{2ass} is the percentage of CO_2 captured in the cathode compartment, CO_{2in} is the amount of CO_2 in the gas mix, and CO_{2ev} is the concentration of CO_2 in the gas mix evolved from the cathode chamber headspace. CO_{2ass} was used to directly measure the percentage of CO_2 captured by each system.

In order to calculate the concentration of CO_2 at saturation in the catholyte, we applied Henry's law equation for gas dissolution in water applicable, with good approximation, to dilute water solutions (with M <1) [4]. Due to the low ionic strength (0.011908 mol/L) and molarity (3.78 mmol/L and 0.164 mmol/L vs. $(NH)_2SO_4$ and $FeSO_4$, respectively), it was possible to refer our calculations to pure water, according to Eq.3 [5]:

$$C = P * K \qquad \textbf{Eq (2S)}$$

where C is the concentration of a gas in the liquid as mol/L, K is Henry's constant for a gas in a solvent (expressed as mol/L atm), and P is the partial pressure of the gas on the solvent, expressed in atm. As we used a gas mix composed of 98% of N_2 and 2% of CO_2, we used Dalton's law to calculate the partial pressure of CO_2 in the mix (Eq. 3S) [4]:

$$P_{tot} = P_{CO2} + P_{N2} \quad \textbf{Eq (3S)}$$

where P_{tot} is the pressure of the gas mix and P_{CO2} and P_{N2} are the partial pressure of CO_2 and N_2, respectively, where partial pressures are given by the total pressure of a gas mix and X, the molar fraction (expressed as percentage) of each component (Eq. 4S):

$$P_{CO2} = P_{tot} * X_{CO2} \quad \textbf{Eq (4S)}$$

Considering a pressure of the gas mix bubbled in the media of 1.2 ± 0.2 atm and the percentage of CO_2 in the mix, P_{CO2}=1.2 atm x 0.02= 0.024 atm at saturation in BESs and microbial cultures headspace. Once we obtained the PCO_2 value, we focused on the calculation of CO_2 dissolved in water. Henry's constant for solubility of a gas in water can be expressed in different ways, e.g., as a function of gaseous solute concentration expressed as mol*(Kg Pa)$^{-1}$ or molarity (M/atm), but even as a function of molar fraction of gas dissolved in the solvent, i.e., water in our experiment. When expressed in the function of the molar fraction, Henry's constant H^{xp} for solubility can be expressed by the following equation Eq (5S) [5]:

$$H^{xp} = X/P. \qquad \textbf{Eq.(5S)}$$

where X is the molar fraction of the gas and P is its partial pressure.

As Henry's constant for solubility is temperature dependent and its values already available in the scientific literature are mostly referred to as 25°C, we applied Eq.6S [4] to calculate H^{xp} of CO_2 at 20°C ($H^{xp\prime}_{CO2}$):

$$\ln\left(\frac{H}{MPa}\right) = -6.8346 + \frac{1.2817x10^4}{T} - \frac{3.7668x10^6}{T^2} + \frac{2.997x10^8}{T^3} \quad \textbf{Eq (6S)}$$

From the resolution of Eq. 7 and the expression of pressure values in atm, $H^{xp\prime}$= 14.13 atm^{-1} [33]. From the application of Eq. 6, X_{CO2} at saturation was

$$X_{CO2} = H^{xp\prime} * P_{CO2} = 14.13\ (atm^{-1}) * 0.024\ atm = 0.339 \quad \textbf{Eq (7S)}$$

Assuming the total amount of moles in the solution is equal to 1, the amount of CO_2 in the catholyte is equal to 0.339 mol. The molarity of CO_2 is given by Eq. (8S):

$$M = XCO_2/V_{cath} \quad \textbf{Eq (8S)}$$

As the volume of the cathode compartment was 0.125 mol, the concentration of CO_2 in the medium was calculated to be 2.7 M. This value represents the amount of CO_2 at saturation, and therefore the initial concentration of CO_2 in our systems. Using the calculated value of $H^{xp'}{}_{CO2}$, we estimated the residual concentration of CO_2 in the catholyte at the end of the experimental session by applying Eqs 5S and 7S.

S3 Carbon conversion efficiency

We calculated the amount of carbon recovery (CR), i.e., the inorganic carbon directly converted in the investigated metabolites and expressed as a % according to Eq. 9S [4] mod:

$$n_{C,t} = \frac{n_{metabolite} * f_{C,metabolite}}{n_{CO2,\Delta t}} * 100 \qquad \text{[Eq. 9S]}$$

where $n_{C,t}$ refers to carbon efficiency at a time t, n is the number of moles of organic compound produced at time t, f c metabolite is the number of moles of carbon in a mole of a given metabolite, and $n_{bic,t0}$ is the number of moles of CO_2 subtracted from the gas mix during the experimental period (6 hours) by each BES, which is calculated as follows:

$$n_{CO2,\Delta t} = n_{CO2\Delta,t,BES} - n_{CO2,\Delta t,blank} \qquad \text{[10S]}$$

As for the control cultures, $n_{C,t}$ is calculated referring to the sterile medium as blank. As the CO_2 in sterile media is captured in consequence of chemical–physical processes, we assumed $n_{CO2,\Delta t}$ can be ascribed to the metabolism of bacteria.

S4 Average well color development (AWCD) calculations and meaning

The capability of microorganisms to utilize different carbon sources was measured by average well color development (AWCD), which assumes that higher carbon source utilization capability corresponds to higher microbial abundance. AWCD is calculated in equation (9S):

$$AWCD = \sum_{i=1}^{n}(C_i - R)/n \quad \textbf{Eq (9S)}$$

where Ci is the absorbance of each reaction well at 590 nm, R is the absorbance of the control well, and n is the number of wells. Values of (*Ci –R*) lower than 0.06 were considered to be zero [4]. AWCD, by interpolating the absorbance linked to microbial growth in each group of wells, provided with a specific carbon substrate, gives a measure of the overall metabolic activity of each tested strain when a specific carbon source is available. From the combination of AWCD values for each tested organic compound, it is possible to determine the metabolic pattern of a test organism.

REFERENCES

1. Junkins, E.N., McWhirter, J.B., McCall, LI. et al. Environmental structure impacts microbial composition and secondary metabolism. ISME COMMUN. 2, 15 (2022). https://doi.org/10.1038/s43705-022-00097-524.

2. Zheng S, Bawazir M, Dhall A, Kim H-E, He L, Heo J and Hwang G (2021) Implication of Surface Properties, Bacterial Motility, and Hydrodynamic Conditions on Bacterial Surface Sensing and Their Initial Adhesion. Front. Bioeng. Biotechnol. 9:643722. doi: 10.3389/fbioe.2021.643722

3.Nastro, R.A., Arguelles-Arias, A., Ongena, M. et al. Antimicrobial Activity of Bacillus amyloliquefaciens ANT1 Toward Pathogenic Bacteria and Mold: Effects on Biofilm Formation. Probiotics & Antimicro. Prot. 5, 252–258 (2013). https://doi.org/10.1007/s12602-013-9143-14. Liu, C., Qin, J., Lin, Y., 2017, 'Fermentation and Redox Potential', in A. F. Jozala (ed.), Fermentation Processes, IntechOpen, London. 10.5772/64640.

5. Bajracharya S., Heijne A., Benetton X.D., Vanbroekhoven K., Buisman C.J.N., Strik D.P.B.T.B, Pant D., Carbon dioxide reduction by mixed and pure cultures in microbial electrosynthesis using an assembly of graphite felt and stainless steel as a cathode, Bioresource Technology, Volume 195, 2015, Pages 14-24, ISSN 0960-8524

6. Dumontet S., Cavoski I., Ricciuti P., Mondelli D., Jarrar M., Pasquale V., Crecchio C. (2017) Metabolic and genetic pat-terns of soil microbial communities in response to different amendments under organic farming system, Geoderma, 296 79-85, ISSN 0016-7061, https://doi.org/10.1016/j.geoderma.2017.02.025.

7. R. Sander Compilation of Henry's law constants (version 4.0) for water as solvent. Atmos. Chem. Phys., 15, 4399–4981, 2015 https://doi.org/10.5194/acp-15-4399-2015

8. John J. Carroll, John D. Slupsky, and Alan E. Mather , "The Solubility of Carbon Dioxide in Water at Low Pressure", Journal of Physical and Chemical Reference Data 20, 1201-1209 (1991) https://doi.org/10.1063/1.555900
